# Green Nanoparticles for Enhanced Electrochemical Monitoring of Pharmaceutical Contaminants: Comparative Investigation Between Monometallic and Bimetallic Nanoparticles

**DOI:** 10.3390/mi17010060

**Published:** 2025-12-31

**Authors:** Soumaya Nasri, Amani Chrouda, Shazalia Mahmoud Ahmed Ali, Bakheit Mustafa, Manahil Babiker Elamin, Laila M. Alhaidari, Hamdi Ben Halima, Nicole Jafezic-Renault

**Affiliations:** 1 Department of Chemistry, Faculty of Science Al-Zulfi, Majmaah University, Majmaah 11952, Saudi Arabia; soumaya.n@mu.edu.sa (S.N.); sm.ahmed@mu.edu.sa (S.M.A.A.); m.elamn@mu.edu.sa (M.B.E.); l.alhaidari@mu.edu.sa (L.M.A.); 2 Department of Chemistry, College of Science, Qassim University, Buraidah 51452, Saudi Arabia; b.salih@qu.edu.sa; 3 UTINAM Institute, UMR CNRS 6213, University Marie et Louis Pasteur, 16 Gray Road, 25030 Besançon, France; hamdi.ben_halima@univ-fcomte.fr

**Keywords:** monometallic nanoparticles, bimetallic nanoparticles, pharmaceutical pollutants, electrochemical measurements

## Abstract

Study presents a comparative analytical investigation into the green synthesis of monometallic and bimetallic nanoparticles using *Punica granatum* (pomegranate) extract, aimed at developing high-performance electrochemical sensors for the detection of ciprofloxacin (CIP) as a representative pharmaceutical pollutant. Three nanoparticle systems were successfully synthesized: monometallic Au@NPs and TiO_2_@NPs, as well as the bimetallic AuTiO_2_@NPs. Their structural and physicochemical characteristics were comprehensively analyzed using UV–Vis spectroscopy, FTIR, SEM, TEM, and XRD techniques. The obtained nanoparticles exhibited predominantly spherical morphologies with average particle sizes of approximately 40 ± 5 nm for Au@NPs, 50 ± 7 nm for TiO_2_@NPs, and 60 ± 6 nm for AuTiO_2_@NPs. These nanomaterials were subsequently employed to modify electrode surfaces for electrochemical sensing applications. Their analytical performance was evaluated using cyclic voltammetry (CV) and square-wave voltammetry (SWV). The sensors displayed excellent sensitivity, with limits of detection of 0.8 ppb for TiO_2_@NPs, 0.8 ppb for Au@NPs, and 0.2 ppb for the AuTiO_2_@NP-based sensor. The bimetallic platform demonstrated superior electrochemical behavior, enhanced signal intensity, and strong selectivity, achieving recovery rates of 98% in tap water and 103% in wastewater. Overall, the results confirm the effectiveness of green-synthesized bimetallic nanoparticles as efficient, low-cost materials for environmental monitoring of emerging pharmaceutical contaminants.

## 1. Introduction

Although water covers approximately 71% of the Earth’s surface, only about 2.5% constitutes freshwater resources that are accessible for essential ecological and human needs [1,2,3]. Among the emerging threats to these limited resources, pharmaceuticals have become a critical global concern, posing then serious risks to both human health and aquatic ecosystems [4]. They enter water bodies through various pathways, including household wastewater, agricultural runoff, and industrial discharges [4]. Due to their chemical stability and resistance to conventional water treatment methods, pharmaceuticals tend to persist in the environment, further complicating efforts to ensure water safety and long-term water security [5]. More dangerous pharmaceutical products, even at trace concentrations, exhibit toxic effects on aquatic organisms, including fish, algae, and invertebrates [6]. Addressing this environmental concern requires the implementation of stricter regulatory frameworks, the development of advanced analytical wastewater monitoring, proper disposal practices, and the promotion of eco-friendly alternatives in pharmaceuticals and personal care formulations.

Compared to conventional techniques for water monitoring, which are often complex, time-consuming, require extensive sample preparation, and depend on skilled staff, electrochemical sensors offer significant advantages, including rapid response, ease of miniaturization, high selectivity, and specificity [7,8]. In particular, electrochemical sensors functionalized with nanomaterials have shown great potential for the effective detection of various contaminants in polluted water sources [9]. This enhanced performance is largely attributed to the unique properties of nanomaterials, such as their high specific surface area, tunable porosity, and superior adsorption and catalytic capabilities [10,11,12,13,14]. These characteristics not only improve the sensitivity and detection limits of the sensors but also contribute to their overall efficiency and practicality in real-world environmental monitoring applications.

A wide range of physical and chemical techniques has been developed for the synthesis of nanoparticles, including magnetron sputtering [15], metal-vapor synthesis [16], chemical reduction [17,18], sonochemical processes [19], and sol–gel methods [20]. While these approaches offer control over particle size and morphology, they often require prolonged reaction times, elevated temperatures, and the use of hazardous chemical reagents, leading to concerns about environmental impact, energy consumption, and potential toxicity [21]. These limitations have highlighted the urgent need for sustainable, eco-friendly synthesis strategies. Green synthesis has emerged as a promising alternative, leveraging natural reducing and stabilizing agents, such as plant extracts, microorganisms, and biopolymers, to produce green bimetallic nanoparticles under mild conditions with minimal environmental footprint [22,23].

Green synthesis nanoparticles involve the concurrent sorption or reduction of metal ions in the presence of suitable reducing and capping agents. Biogenic materials, including plant extracts, biomolecules, including proteins, carbohydrates, and nucleic acids, are considered ideal green reagents due to their relative safety, low cost, and ease of use [21,22,23,24].

While monometallic nanoparticles have demonstrated good performances, combining two different metals could probably offer clear advantages that can significantly boost detection and remediation efficiency [24,25]. These nanomaterials are in fact able to provide: (i) greater stability and resistance to agglomeration and oxidation because the combination of metals stabilizes the nanoparticle structure, preventing surface degradation and extending their useful life [26,27]; (ii) synergistic interaction between the two metals enhances adsorption and catalytic performance, surpassing what monometallic particles can achieve [26,27]: (iii) specific interaction with different surface compositions and electronic properties, enabling selective targeting of pollutants while minimizing interference from other substances [28,29]. Their versatile composition, morphology, and surface chemistry make them highly adaptable for different environmental conditions [30]. Different sensors based on green bimetallic nanoparticles have already explored for several analyte detection. A simple method was developed for the green synthesis of bimetallic silver-palladium nanoparticles (BG-AgPd-NPs) using bael gum (Aegel marmelos). The designed bimetallic nanoparticles have shown a potential for lead ion detection with a limit of detection (LOD) inferior to 1 µM [31]. Ag-Au bimetallic nanoparticles (Ag-AuNPs) supported on reduced graphene oxide (RGO) with alginate as reductant and stabilizer were also designed. The peroxidase-based sensor demonstrated excellent performance in detecting H_2_O_2_, with a linear range of 0.1 to 10 mM, and a low detection limit of 0.57 µM [32]. In other investigations, silver-platinum (Pt-Ag) bimetallic nanoparticles were synthesized by a biogenic reduction method using plant extracts. The sensor limit of detection (LOD) was 0.03 µM, and the limit of quantification (LOQ) was 0.11 µM [33]. One work used green-synthesized silver nanoparticles and carbon black for the electrochemical detection of ciprofloxacin. A detection limit of 0.48 µM [34].

However, to the best of our knowledge, no previous study has compared the performances of monometallic and bimetallic nanoparticles for the detection of pharmaceutical pollutants. For these reasons, the present work reports a comparative investigation of sensors functionalized with green monometallic and bimetallic nanoparticles using *Punica granatum* (pomegranate) extract, for ciprofloxacin (CIP) detection, which is employed here as a model pharmaceutical contaminant. Three types of nanoparticles were then synthesized: Au@NPs, TiO_2_@NPs, and AuTiO_2_@NPs. The successful synthesis and characterization of these nanoparticles were confirmed using various analytical techniques, including UV-Vis spectroscopy, Fourier-transform infrared spectroscopy (FTIR), transmission electron microscopy (TEM), and X-ray diffraction (XRD). Furthermore, the modification and the analytical performances of the electrochemical sensor surface were evaluated using cyclic voltammetry (CV) and square wave voltammetry (SWV).

## 2. Materials and Methods

### 2.1. Products

All chemicals and reagents were of analytical grade and used as received without further purification. Titanium dioxide (TiO_2_, ≥99%), hydrogen tetrachloroaurate (III) (HAuCl_4_, ≥99.9%), potassium ferricyanide (III) (K_3_[Fe(CN)_6_], ≥99%), potassium ferrocyanide (II) (K_4_[Fe(CN)_6_], ≥99%), and buffer components including phosphate-buffered saline (PBS, pH 7.4), acetate buffer, borate buffer, and Tris buffer (tris(hydroxymethyl)aminomethane, ≥99%) were purchased from Sigma-Aldrich, St. Louis, MO, USA. The antibiotics ciprofloxacin (≥98%), norfloxacin (≥98%), amoxicillin (≥98%), tetracycline (≥98%), sulfamethoxazole (≥98%), and chloramphenicol (≥98%) were also obtained from Sigma-Aldrich.

### 2.2. Instrumentation

The UV-Visible spectra were recorded with a Perkin Elmer (Sprinfield, IL, USA) Lambda 950 UV spectrophotometer, scanning wavelengths from 200 to 800 nm with a resolution of 1 nm, with samples in chloroform at room temperature. The Fourier Transform Infrared Spectroscopy (FTIR) spectra of the *Punica granatum* extract (PP), Titanium Nanoparticles TiO_2_@NPs, gold Nanoparticles Au@NPs, and the gold Titanium Nanoparticles AuTiO_2_@NPs were recorded in the 4000–400 cm^−1^ domain using a Perkin Elmer FT-IR spectrophotometer. Transmission electron microscopy (TEM) was performed using a high-resolution TEM instrument (e.g., JEOL 2100F, (Peabody, MA, USA)), with an acceleration voltage of 200 kV, ensuring detailed imaging at the nanometer scale. To prepare the samples for TEM analysis, a small drop of the nanoparticle dispersion was deposited onto a copper grid coated with a thin carbon film and dried under ambient conditions. X-ray diffraction was carried out using a Bruker (Allentown, PA, USA) D8 Advance polycrystalline powder/X-ray diffractometer (PXRD) equipped with a Cu X-ray source (wavelength = 1.5406 Å) in Bragg–Brentano geometry operating at 40 mA and 40 kV.

### 2.3. Nanoparticle Synthesis

**Scheme 1:**
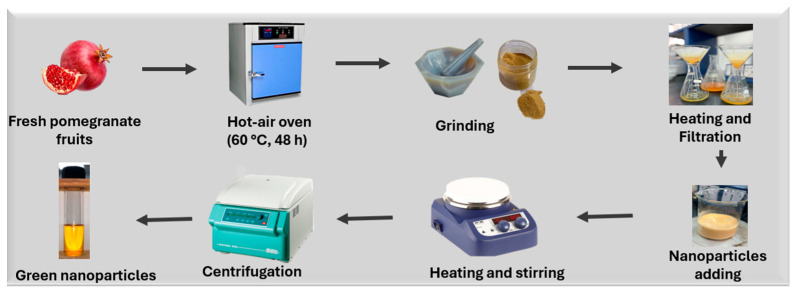
Green synthesis of nanoparticles using *Punica granatum* green extract.

This study employed a green synthesis approach for the fabrication of metal and metal oxide nanoparticles using *Punica granatum* peel extract as a natural reducing and stabilizing agent. A schematic representation of the overall concept of green nanoparticle synthesis is presented in Scheme 1.

#### 2.3.1. Preparation of *Punica granatum* Peel Extract

Fresh pomegranate fruits were obtained locally and thoroughly rinsed with distilled water. The peels were manually separated from the fruit pulp and cut into small pieces to facilitate drying. The peel fragments were then dried in a hot-air oven at 60 °C for 48 h to ensure complete dehydration while preserving bioactive compounds. Once dried, the peels were ground into a fine powder using a mechanical grinder and passed through a sieve to achieve a uniform particle size. For extract preparation, 5 g of this powder was mixed with 100 mL of distilled water in a beaker. The mixture was heated at 100 °C for 5 min to extract phytochemicals such as polyphenols, flavonoids, and tannins, which are known to play a role in nanoparticle formation. After heating, the solution was allowed to cool to room temperature and filtered using Whatman No. 1 filter paper. The resulting clear yellow extract was stored at 4 °C and used within 24 h to ensure activity. All extraction processes were carried out under ambient laboratory conditions (~25 °C, 45–55% relative humidity), without any need for an inert atmosphere.

#### 2.3.2. Coating of Titanium Dioxide Nanoparticles (TiO_2_@NPs)

Titanium dioxide nanoparticles were coated with the previously prepared *P. granatum* extract. Specifically, 0.04 g of TiO_2_ powder was gradually added to 100 mL of the extract under magnetic stirring to ensure uniform dispersion. The reaction mixture was maintained at 80 °C and continuously stirred for 4 h. During this period, a gradual color change in the solution was observed, indicating nanoparticle formation. The phytochemicals in the extract facilitate the reduction and stabilization of TiO_2_ nanoparticles by acting as both capping and reducing agents. After the reaction, the mixture was allowed to cool, then centrifuged at 8000 rpm for 10 min to collect the solid nanoparticles. The resulting pellet was washed three times with distilled water to remove unreacted biological residues and dried in an oven at 80 °C until a constant weight was achieved. The final product, referred to as TiO_2_@NPs, was stored in airtight containers for further characterization.

#### 2.3.3. Synthesis of Gold Nanoparticles (Au@NPs)

Gold nanoparticles were synthesized in the presence of *Punica granatum* peel extract. A solution of 0.08 g of hydrogen tetrachloroaurate (III) (HAuCl_4_) was prepared in 100 mL of the *Punica granatum* peel extract. The mixture was stirred and heated at 80 °C for approximately 30 min. A color transition from light yellow to deep purple was observed, confirming the formation of gold nanoparticles due to the localized surface plasmon resonance (LSPR) effect. This visual change serves as a preliminary indicator of successful nanoparticle synthesis. After the reaction, the solution was cooled and centrifuged at 9000 rpm for 15 min. The pellet containing Au@NPs was washed repeatedly with deionized water to eliminate excess plant biomolecules and dried in an oven at 80 °C. The obtained nanoparticles were stored in a desiccator until use. All synthesis steps were conducted under ambient pressure and atmospheric conditions without inert gas protection, further confirming the green and accessible nature of the method.

#### 2.3.4. Synthesis of Gold-Supported Titanium Dioxide Nanoparticles (AuTiO_2_@NPs)

To prepare bimetallic nanoparticles with enhanced physicochemical properties, gold nanoparticles were supported on titanium dioxide using a hybrid green synthesis protocol. In a 1:1:1 volume ratio, 50 mL of 1.5 mM HAuCl_4_ solution, 50 mL of *Punica granatum* peel extract, and 50 mL of distilled water were mixed in a round-bottom flask and stirred at 60 °C for 1 h to initiate the reduction of gold ions. The gradual appearance of a purple hue indicated successful AuNP formation. Subsequently, 500 mg of pre-synthesized TiO_2_@NPs were added to the reaction mixture, which was then subjected to continuous stirring at 200 °C for an additional 2 h in a sealed system to promote the loading of gold onto the TiO_2_ surface. After completion, the resulting AuTiO_2_@NPs were separated by centrifugation at 10,000 rpm, washed multiple times with a 1:1 methanol–distilled water solution to remove unbound species and residual phytochemicals, and air-dried at room temperature under ambient conditions. This method required moderate thermal energy and avoided the use of organic solvents, toxic reducing agents, or complex equipment, making it eco-friendly and scalable.

### 2.4. Electrochemical Sensor Design

A screen-printed gold electrode (SPGE) purchased from Metrohm Dropsens, Herisau, Switzerland, including a gold working electrode (diameter: 4 mm) integrated with a platinum auxiliary electrode and an Ag/AgCl pseudo-reference electrode, was employed for the sensor fabrication. Before modification, the electrode surface was thoroughly cleaned by sequential rinsing with absolute ethanol and distilled water, followed by drying under ambient conditions. After that, the surface was modified with the different prepared nanoparticles, separately.

Electrochemical characterization and measurements were conducted at room temperature using a potentiostat system (Ivium Technologies, Eindhoven, The Netherlands) housed in the Department of Chemistry, Faculty of Science, Majmaah University. Cyclic voltammetry (CV) was utilized to assess the electrochemical properties of the modified sensing surfaces in the presence of a standard redox couple, 5 mM [Fe(CN)_6_]^3−/4−^ prepared in 0.1 M phosphate-buffered saline (PBS, pH 7.4). Following surface characterization, square-wave voltammetry (SWV) was employed for quantitative analysis. SWV parameters were: initial potential: −0.1 V; end potential: +0.5 V; step potential: 0.01 V; modulation amplitude: 0.05 V. The sensor was incubated in varying concentrations of ciprofloxacin, and SWV was subsequently performed to construct the calibration curve, enabling evaluation of the sensor’s sensitivity and dynamic detection range.

For the electrochemical measurements, all experiments were repeated three times to ensure the reproducibility of the results.

## 3. Results and Discussion

### 3.1. Nanoparticles Synthesis

#### 3.1.1. UV-Spectroscopy

**Figure 1:**
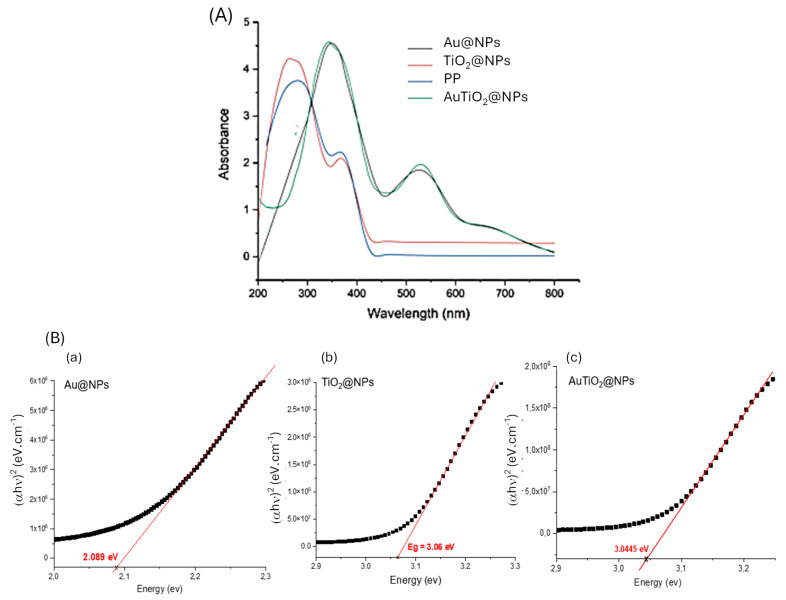
(**A**) UV Visible spectra of *Punica granatum* (PP), Au@NPs, TiO_2_@NPs, and AuTiO_2_@NPs in the wavelength range of 200–800 nm; and (**B**) Tauc plot of the (αhν)^2^ versus hν plots: (**a**) Au@NPs, (**b**) TiO_2_@NPs, and (**c**) AuTiO_2_@NPs.

To confirm the successful synthesis of Au@NPs, TiO_2_@NPs, and AuTiO_2_@NPs, UV-Visible spectroscopy was performed over a wavelength range of 200–800 nm. The UV-Vis absorption spectrum of *Punica granatum* peel extract, shown in Figure 1A, reveals a prominent peak at 280 nm and a secondary peak at 370 nm. These absorption features are attributed to the rich presence of bioactive compounds in the *Punica granatum* peel extract, including flavonoids (such as flavanols and anthocyanins), condensed tannins (e.g., proanthocyanidins), and hydrolysable tannins (e.g., ellagitannins and gallotannins), all of which contribute to strong absorbance in this spectral region. The observed peaks are consistent with those reported in the literature for *Punica granatum* peel extracts, validating the extract’s composition [35,36].

Following the synthesis of TiO_2_@PP nanoparticles, a spectral shift and an increase in absorbance intensity were observed, indicating a successful nanoparticle coating. Polyphenols, flavonoids, and other phytochemicals in the extract act as in fact as coating and stabilizing agents, adsorbing onto the nanoparticle surface through hydrogen bonding or coordination with surface titanium atoms. These interactions prevent particle agglomeration, enhance colloidal stability, and can slightly alter the electronic environment of TiO_2_, leading to the observed spectral shifts. These findings align with those reported by H. M. Abdelmigid et al. [37], suggesting that the extract efficiently facilitates the stabilization of metal oxide. For the Au@NPs, the UV-Vis spectrum exhibited a characteristic band around 370 nm, which may be attributed to phytochemicals from *Punica granatum*, and a distinct band at 530 nm corresponding to the LSPR of gold nanoparticles, thereby confirming their successful formation. The LSPR peak is in agreement with previous studies by S. Bawazeer et al. [38] and S. S. Dash et al. [39]. Notably, the UV-Vis spectrum of the bimetallic AuTiO_2_@NPs displayed similar absorption characteristics to that of AuTiO_2_, reflecting the combined properties of the gold and titanium oxide components in the nanoparticle structure.

To provide insight into the material’s potential functionality and its suitability for targeted electrochemical applications, an accurate determination of the band gap is essential. The optical band gap energy of the synthesized nanoparticles was estimated based on UV-Vis spectroscopy measurements using the Tauc method [40], as described by the following equation:

(αhν)^n^ = A(hν − Eg) (1)

In the given equation, α: absorption coefficient, hν: photon energy, Eg: band gap energy; *n*: constant depending on the type of transition (typically 2 for direct allowed, ½ for indirect allowed transitions), and A: a constant.

The band gap was determined by extrapolating the linear region of the Tauc plot of the (αhν)^2^ versus hν plots, as illustrated in Figure 1B. The resulting computations revealed that the Au@NPs, TiO_2_@NPs, and AuTiO_2_@NPs exhibited band gap energies of 2.089 eV, 3.06 eV, and 3.044 eV, respectively. The variation in the band gap energies for different nanoparticles could be attributed to differences in their composition and electronic interactions. The relatively low band gap of Au@NPs is associated with the localized surface plasmon resonance (LSPR) and interband transitions characteristic of metallic gold nanoparticles, which do not possess a conventional semiconductor band gap [41]. In contrast, TiO_2_@NPs exhibit a band gap of 3.06 eV, consistent with the anatase phase of TiO_2_, a wide bandgap semiconductor known for its strong UV absorption [42].

The slight reduction in the band gap for AuTiO_2_@NPs (3.044 eV) compared to pure TiO_2_ suggests a synergistic interaction between the gold and TiO_2_ components. This interaction results from electronic coupling at the Au and TiO_2_ interface, where gold nanoparticles can facilitate electron transfer from the conduction band of TiO_2_ to the electrode and enhance light absorption through plasmonic effects. These results are in good agreement with those found by B. Y. Balarabe [43].

#### 3.1.2. FTIR Characterization

**Figure 2:**
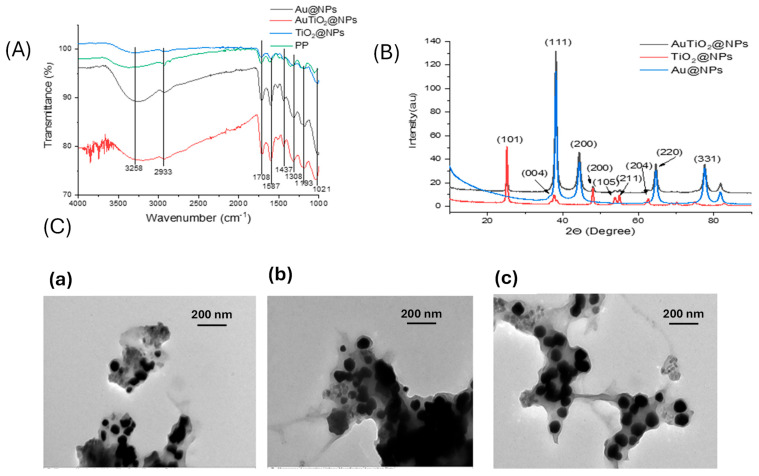
(**A**) FTIR spectra of *Punica granatum* (PP), Au@NPs, TiO_2_@NPs and AuTiO_2_@NPs in the range of wavenumber varied from 1000 to 4000 cm^−1^; (**B**) XRD of Au@NPs, TiO_2_@NPs and AuTiO_2_@NPs; (**C**) TEM spectra of: (**a**) Au@NPs; (**b**) TiO_2_@NPs; and (**c**) AuTiO_2_@NPs.

Fourier-transform infrared (FTIR) spectroscopy was employed to identify the functional groups present in the samples based on their characteristic vibrational frequencies. All four FTIR spectra exhibited similar profiles, suggesting the presence of comparable functional groups across the samples, which are attributed to the phytochemical constituents of *Punica granatum* (Figure 2A). A broad absorption band around 3258 and 2936 cm^−1^ is attributed to O–H stretching vibrations from carboxylic acids, as well as C–H stretching vibrations typical of alkanes. A peak observed near 1708 cm^−1^ indicates the presence of C=O stretching vibrations, suggesting carbonyl-containing groups such as ketones, aldehydes, and carboxylic acids. The band at 1587 cm^−1^ is associated with C=C stretching in aromatic rings and N–H bending vibrations of primary amines. A distinct peak at 1437 cm^−1^ corresponds to C–H rocking in alkanes and may also indicate symmetric N=O stretching in nitro compounds. Absorption at 1308 cm^−1^ is characteristic of C–O stretching, typically found in alcohols, carboxylic acids, esters, and ethers. Additionally, a band at 1193 cm^−1^ is indicative of C–N stretching vibrations in aliphatic amines. A peak around 1021 cm^−1^ can be attributed to N–H wagging in primary and secondary amines, as well as C–H out-of-plane bending in aromatic compounds.

The observed functional groups are consistent with those reported in previous studies, confirming the presence of various organic compounds derived from *Punica granatum* [22,44]. The enhanced FTIR absorption intensity observed in the nanoparticle samples, compared to the *Punica granatum* extract alone, can be attributed to an increase in the vibrational dipole moment of these functional groups following nanoparticle synthesis. This enhancement results from strong interactions between the phytochemicals and the nanoparticle surfaces, leading to more pronounced IR-active vibrations, especially in the case of bimetallic nanoparticles.

#### 3.1.3. X-Ray Diffraction

For evaluating the crystalline and phase structures of the prepared nanomaterials, Figure 2B presents the X-ray diffraction (XRD) pattern of the synthesized TiO_2_ nanoparticles. The diffraction analysis revealed well-defined peaks at 2θ values of 25.5°, 37.7°, 48.1°, 53.8°, 54.9°, and 62.5°, which correspond to the (101), (004), (200), (105), (211), and (204) crystallographic planes, respectively. These reflections are in good agreement with the standard diffraction data for the anatase phase of TiO_2_, as reported in previous studies [45] and confirmed by the Joint Committee on Powder Diffraction Standards (JCPDS) card No. 84-1285. The dominance of the (101) peak indicates that this plane is the most thermodynamically stable and preferentially oriented during crystal growth. The absence of peaks related to the rutile or brookite phases suggests high phase purity of the anatase TiO_2_. This phase is particularly desirable due to its higher surface area and better charge carrier mobility. The XRD pattern of Au@NPs displays characteristic diffraction peaks at 2θ values of 38.2°, 44.3°, 64.2° and 77.9°, corresponding to the (111), (200), and (331) planes of face-centered cubic gold (Au), in accordance with the JCPDS card no. 04–0784. The XRD spectrum of AuTiO_2_@NPs exhibits all the diffraction peaks associated with Au@NPs, along with those of TiO_2_@NPs, confirming the formation of a composite material. The presence of distinct diffraction peaks from both Au and TiO_2_ indicates the coexistence of their respective crystalline phases in the final AuTiO_2_@NPs nanostructure, at the same angle as in the pure phase. These results confirm the successful incorporation of Au nanoparticles into the TiO_2_ matrix, in agreement with the findings reported by Z. Duan et al. [46].

#### 3.1.4. Transmission Electron Microscopy (TEM) and Scanning Electron Microscopy (TEM)

The TEM image of Au@NPs (Figure 2C(a)) reveals well-dispersed, spherical gold nanoparticles with an average particle diameter of approximately 40 ± 5 nm. The particles exhibit a narrow size distribution and are predominantly monodisperse, confirming the high degree of uniformity achieved during synthesis. *Punica granatum* extract improves nucleation, avoids agglomeration, and improves stability, as already observed in green synthesis in the presence of other extracts [47].

In contrast, the TEM image of TiO_2_@NPs (Figure 2C(b)) displays spherical nanoparticles with a slightly broader size distribution compared to the gold nanoparticles, with an average diameter of 50 ± 7 nm. Although the TiO_2_ nanoparticles are generally uniform in shape, a slight degree of aggregation is observed, which is typical for TiO_2_-based materials. The distinct contrast between the particles and the background confirms the crystalline nature of TiO_2_. Furthermore, the smooth surfaces of the particles suggest the predominance of the anatase phase, consistent with the XRD results.

The TEM image of AuTiO_2_@NPs (Figure 2C(c)) reveals a composite nanostructure with an average particle size of 60 ± 6 nm, where gold nanoparticles are closely integrated within the TiO_2_ matrix, forming a core–shell or hybrid configuration. This morphology indicates the successful incorporation of Au into the TiO_2_ framework. No significant aggregation of either component is observed, demonstrating the good stability and dispersion of the composite.

### 3.2. Sensor Design

#### 3.2.1. Surface Characterization

**Figure 3:**
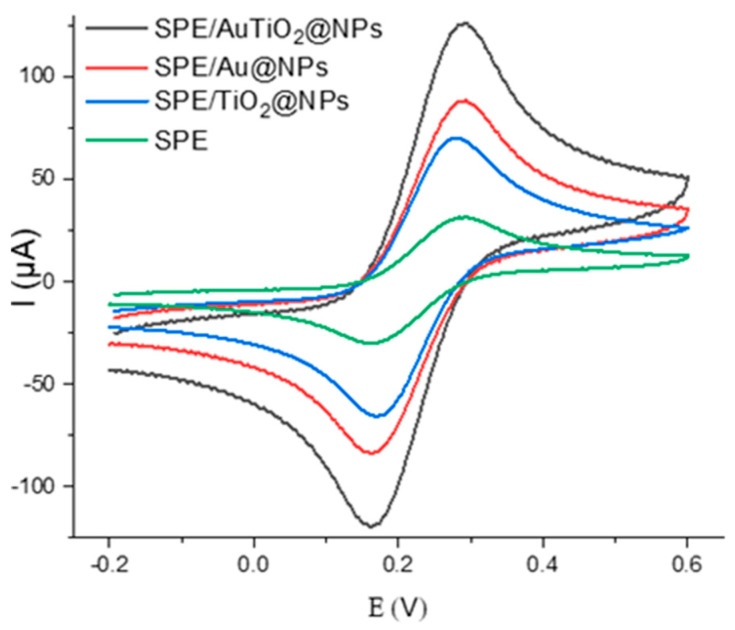
Cyclic voltammetry voltammograms of the gold surface before and after modification with Au@NPs, TiO_2_@NPs, and AuTiO2@NPs. All measurements are performed in 5 mM of [Fe(CN)_6_]^3−^/^4−^ in PBS buffer (0.1 M).

Building on the physicochemical properties of the synthesized nanomaterials of Au@NPs, TiO_2_@NPs, and AuTiO_2_@NPs nanoparticles and to ensure a more comparative investigation concerning the properties of monometallic and bimetallic nanoparticles in the enhancement of electrochemical signal, three different electrochemical sensors were developed by functionalizing screen-printed electrodes (SPGEs) with these nanomaterials. The electrochemical behavior of the electrodes, both before and after surface modification, was evaluated in a 0.1 M phosphate-buffered saline (PBS, pH 7.4) solution containing 5 mM of the redox couple [Fe(CN)_6_]^3−^/^4−^. To assess the electron transfer capability and electrical conductivity of the modified electrodes, cyclic voltammetry (CV) was performed (Figure 3). The cyclic voltammograms revealed an increase in peak current for the different surface modifications with the green nanoparticle electrode compared to the bare SPGE, indicating enhanced electron transfer kinetics.

Electrochemical measurements reveal that the peak current follows the trend AuTiO_2_@NPs > Au@NPs > TiO_2_@NPs, the bimetallic AuTiO_2_ composite exhibiting the highest electrochemical response. This enhancement can be explained by the synergistic mechanism between gold and titanium dioxide at the nanoscale. Gold nanoparticles provide high electrical conductivity and excellent catalytic activity, which accelerate electron transfer [48].

#### 3.2.2. Effect of the Supporting Electrolyte on the Electrochemical CIP Signal

To enhance the output signal of CIP oxidation, various buffers were tested, including PBS, acetate, borate, and Tris, alongside surface modifications using Au@NPs, TiO_2_@NPs, and AuTiO_2_@NPs. The results indicate that the electrochemical response of CIP is significantly superior when the surface is modified with AuTiO_2_@NPs across all tested electrolytes. This enhancement can be attributed to the synergistic effects between gold and titanium dioxide at the nanoscale. Gold nanoparticles (AuNPs) contribute high electrical conductivity and excellent catalytic activity, enabling rapid electron transfer. On the other hand, TiO_2_, as a semiconductor, exhibits a strong adsorption capacity for polar and charged molecules like CIP, particularly through surface hydroxyl groups that facilitate hydrogen bonding and electrostatic interactions. Among the different surface modifications tested, PBS emerges as the optimal supporting electrolyte for electrochemical CIP detection. CIP, an amphoteric fluoroquinolone antibiotic, exhibits pH-dependent electrochemical behavior, with its electroactive form predominating near physiological pH (6.5–7.5) [49,50,51]. PBS maintains a stable pH in the range of 7.0–7.4, where both ionizable groups of CIP, the carboxyl and piperazinyl moieties, are optimally protonated/deprotonated to contribute to well-defined redox activity, thereby enhancing detection sensitivity. In contrast, other buffers such as acetate (pH 3.6–5.6) tend to protonate CIP excessively, suppressing its redox response [49,50,51]. Borate buffer (pH 8.0–10.0), on the other hand, may lead to full deprotonation of CIP and altered adsorption behavior on the sensor surface [49,50,51]. Tris buffer is also less suitable, as it is known to interact with metal oxide surfaces and can inhibit electron transfer [52]. PBS, by comparison, is electrochemically inert and does not interfere with the active sites of Au or TiO_2_. Its high ionic strength supports efficient charge transport, while its chemical stability minimizes surface passivation. PBS was then selected for CIP detection.

#### 3.2.3. CIP Detection

**Figure 4:**
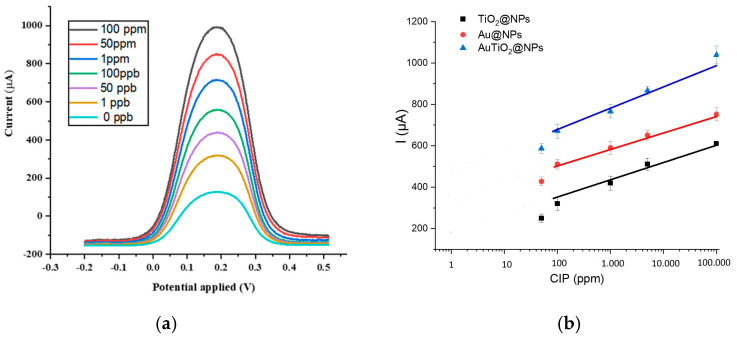
(**a**) SWV voltammograms variation versus different CIP concentrations for the sensor functionalized AuTiO_2_@NPs. Measurements are recorded in PBS buffer (pH = 7.4), and (**b**) Current variation versus different concentration variation for the sensor functionalized Au@NPs, TiO_2_@NPs, and AuTiO_2_@NPs.

The sensing performance of various sensors toward ciprofloxacin (CIP) was evaluated using square wave voltammetry (SWV). The sensors were incubated in CIP solutions at different concentrations for 30 min before measurement. To avoid overcrowding the figure, we present only the SWV voltammograms recorded at varying CIP concentrations in the case of a sensor functionalized with AuTiO_2_@NPs, as the oxidation profiles for CIP were similar across the different sensors. All measurements were performed in triplicate, showing a relative standard deviation (RSD) below 5%. An electrochemical signal for CIP was observed at 0.18 V (Figure 4a) [53].

As the CIP concentration increased, a corresponding rise in the oxidation current was recorded.

For better visualization, the current intensity variation for different sensors versus different CIP concentrations is presented in Figure 4b. A linear relationship was observed in the case of the different sensors, demonstrating the sensor’s capability for quantitative detection. Limits of detection (LOD), linear range, and sensitivity values for the different sensors are presented in Table 1.

The combination of two metallic nanoparticles enhances the electrical conductivity and electrochemical activity of the sensor, leading to a lower LOD and higher sensitivity compared to the individual components.

The sensors developed in this study (Au@NPs, TiO_2_@NPs, and AuTiO_2_@NPs) exhibit highly competitive performance compared to previously reported systems: large linear dynamic range and low detection limits, the SPGE/AuTiO_2_@NPs showing the most promising analytical characteristics (Table 2).

#### 3.2.4. Stability and Repeatability

The operational and storage stability of the modified electrodes was systematically investigated. Operational stability was examined through repeated electrochemical measurements, where the AuTiO_2_@NP-based sensor maintained over 95% of its initial current response after 20 continuous measurement cycles, demonstrating strong resistance to surface fouling and signal degradation. Long-term stability was evaluated by storing the modified electrodes at room temperature and periodically recording their electrochemical response toward CIP. After 14 days of storage, the sensor retained approximately 92% of its original response, indicating satisfactory stability and durability. The good stability can be attributed to the strong interaction between the bimetallic nanoparticles and the electrode surface, as well as the stabilizing effect of the *Punica granatum* extract used during green synthesis.

The repeatability of the developed electrochemical sensors was evaluated by performing consecutive measurements of ciprofloxacin (CIP) at a fixed concentration under identical experimental conditions using the same modified electrode. For the AuTiO_2_@NP-modified electrode, the peak current response showed minimal variation over 20 successive SWV measurements, retaining more than 95% of the initial signal intensity. The relative standard deviation (RSD) of the recorded peak currents was below 3.5%, indicating excellent repeatability and reliable sensor response. To further assess fabrication reproducibility, five independently prepared AuTiO_2_@NP-modified electrodes were tested toward CIP detection. The obtained current responses exhibited good consistency, with an RSD of less than 4%, confirming the robustness of the electrode modification procedure.

#### 3.2.5. Selectivity Measurements

**Figure 5:**
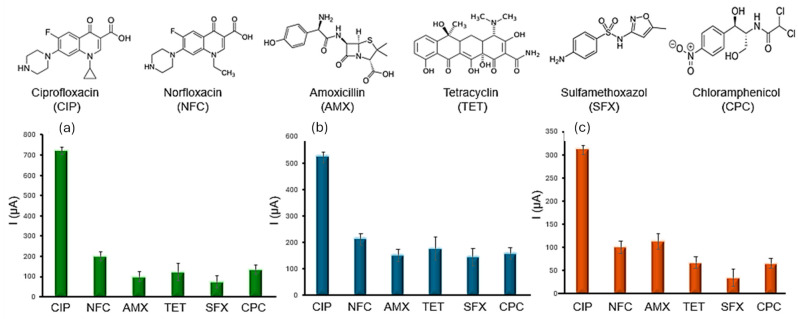
Sensor selectivity measurements: (**a**) Response toward interfering antibiotics for the sensor modified with Au@NPs; (**b**) Response toward interfering antibiotics for the sensor modified with TiO2@NPs; (**c**) Response toward interfering antibiotics for the sensor modified with AuTiO2@NPs. All measurements were recorded in PBS buffer (pH 7.4) at a fixed concentration of 1 ppb for each molecule.

The selectivity of the Au@NPs, TiO_2_@NPs, and AuTiO_2_@NPs sensors against antibiotics such as norfloxacin (similar to ciprofloxacin), amoxicillin, tetracycline, sulfamethoxazole, and chloramphenicol was evaluated at a fixed concentration of 1 ppb (Figure 5). The sensors exhibited good selectivity, with the AuTiO_2_@NPs-functionalized sensor. Norfloxacin, a quinolone antibiotic, differs from ciprofloxacin primarily in the side chain at position 7 (piperazine in ciprofloxacin vs. a methyl group in norfloxacin) and the presence of a cyclopropyl group in ciprofloxacin. These structural differences result in norfloxacin having a relatively higher oxidation potential compared to ciprofloxacin, which enhances the sensor’s selectivity towards CIP in the applied potential range.

Amoxicillin (a β-lactam antibiotic), tetracycline, sulfamethoxazole, and chloramphenicol (aromatic structures with nitrogen-containing groups) require higher potentials for detection. Overall, the different sensors, particularly the AuTiO_2_@NPs sensor, demonstrate the highest selectivity and sensitivity across a broad range of antibiotics. This enhanced performance is attributed to the combined catalytic and conductive properties of the AuTiO_2_@NPs, making it the most efficient platform for the electrochemical sensing of ciprofloxacin (CIP).

#### 3.2.6. Validation in Real Samples

Table 3 compares the analytical performance of the three nanoparticle-modified sensors, Au@NPs, TiO_2_@NPs, and AuTiO_2_@NPs, based on recovery rates and reproducibility (RSD%) for ciprofloxacin detection in tap water and wastewater at three concentration levels. In tap water, the bimetallic AuTiO_2_@NPs sensor demonstrated the most consistent and accurate recovery values across all concentrations (98%) with low RSDs (1.1–3.8%), indicating excellent analytical precision and matrix tolerance. In a more complex matrix such as wastewater, the AuTiO_2_@NPs sensor again maintained strong performance, achieving recoveries between 101% and 108% with RSDs below 4%, indicating good robustness under real-world conditions.

Bimetallic AuTiO_2_@NPs, combining high accuracy, excellent reproducibility, and low matrix effect, confirm the synergistic advantage of the green combination of hybrid nanomaterial in electrochemical detection of ciprofloxacin.

## 4. Conclusions

The present study provides a pioneering comparative investigation into the green synthesis of monometallic (Au@NPs, TiO_2_@NPs) and bimetallic (AuTiO_2_@NPs) nanoparticles using *Punica granatum* extract, with a focus on their application in the electrochemical detection of ciprofloxacin (CIP) as a model pharmaceutical contaminant. Electrochemical studies using cyclic voltammetry (CV) and square wave voltammetry (SWV) demonstrated that all nanoparticle-modified electrodes significantly enhanced the analytical response toward CIP compared to the bare SPGE. The sensors exhibited excellent performance, with a lower limit of detection for AuTiO_2_@NPs (LOD = 0.2 ppb). The superior performance of the bimetallic AuTiO_2_@NPs is attributed to the synergistic combination of gold’s high conductivity and catalytic efficiency with TiO_2_’s large surface area and adsorption capability. Importantly, the sensors demonstrated high selectivity and reliable performance in real water samples, highlighting their practical utility for trace-level pharmaceutical monitoring. Overall, these findings emphasize the potential of green-synthesized bimetallic nanoparticle platforms as sensitive, sustainable, and cost-effective tools for environmental electrochemical sensing. Future work could explore extending this green synthetic strategy to other metal or metal oxide systems and integrating these materials into portable, on-site devices for real-time environmental monitoring.

## Figures and Tables

**Table 1:** Sensitivities, linear ranges and LODs of Au@NPs, TiO_2_@NPs, and AuTiO_2_@NPs based sensors.

Nanomaterial	Sensitivity (µA/ppm)	Linear Range (µM)	LOD	R^2^
**TiO_2_@NPs**	79.69 ± 6.63	3 × 10^−1^ to 3 × 10^2^	0.8 ppb 2.4 × 10^−9^ M,	0.97
**Au@NPs**	83.39 ± 6.39	3 × 10^−1^ to 3 × 10^2^	0.8 ppb 2.4 × 10^−9^ M	0.97
**AuTiO_2_@NPs**	102.96 ± 6.39	3 × 10^−1^ to 3 × 10^2^	0.2 ppb 0.64 × 10^−9^ M	0.98

**Table 2:** Comparison with previously published electrochemical sensors for ciprofloxacin detection (receptor type, linear range, and LOD).

Receptor Nature	Receptor	Range (µM)	LOD (nM)	Refs
**Biological receptors**	dsDNA	0.1 to 100	10^2^	[54]
**Molecularly imprinted polymer**	MIP/rGO/GCE	0.001 to 0.5	0.052	[55]
**Chemical nanomaterials**	AuNPs/AC/GCE	0.5 × 10^3^ to 25× 10^3^	0.20	[56]
	eCNF/CNT/NiCo/GCE	-	6.0 × 10^3^	[57]
	Ca_2_CuO_3_NS/GCE	0.05 to 0.8	0.012	[58]
	TiO_2_/PVA/GCE	10 to 120	40	[59]
	EGr/GC	-	1	[60]
	V_2_O_5_/SPE	-	0.01	[61]
	ERGO/PANI/PARS/SPCE	0.01 to 69.8	2.1	[62]
**Green nanomaterials**	AgNPs/C black/GCE	3 × 10^3^–1.3 × 10^5^	480	[34]
	TiO_2_@ NPs/SPE	3 × 10^−1^ to 3 × 10^2^	2.41	The present work
	Au@NPs/SPE		1.81	
	AuTiO_2_@ NPs/SPE		0.64	

**Table 3:** Sensor’s validation in real samples.

	Sensor Nature	Spiked Concentration (ppm)	Recovered Concentration (ppm)	Recovery (%) (*n* = 3)	RSD (%)
**Tapwater**	Au@NPs	50	47.2	**95**	**1.8**
		100	89.9	**90**	**3.3**
		500	479.1	**96**	**1.6**
	TiO_2_@NPs	50	52.4	**105**	**1.8**
		100	110.5	**116**	**3.3**
		500	520.3	**105**	**2.1**
	AuTiO_2_@NPs	50	49.1	**98**	**3.8**
		100	98.4	**98**	**1.3**
		500	489.9	**98**	**1.1**
**Wastewater**	Au@NPs	50	51.4	**103**	**1.7**
		100	108.5	**109**	**2.2**
		500	512.3	**102**	**1.6**
	TiO_2_@NPs	50	53.4	**107**	**1.0**
		100	109.5	**110**	**2.9**
		500	514.3	**103**	**1.1**
	AuTiO_2_@NPs	50	50.4	**101**	**1.8**
		100	107.5	**108**	**3.9**
		500	511.3	**102**	**3.6**

## Data Availability

The original contributions presented in this study are included in the article. Further inquiries can be directed to the corresponding author.
